# Spectacular alterations in the female reproductive system during the ovarian cycle and adaptations for matrotrophy in chernetid pseudoscorpions (Pseudoscorpiones: Chernetidae)

**DOI:** 10.1038/s41598-022-10283-z

**Published:** 2022-04-19

**Authors:** Arnold Garbiec, Jana Christophoryová, Izabela Jędrzejowska

**Affiliations:** 1 Department of Animal Developmental Biology, Faculty of Biological Sciences, University of Wrocław, Sienkiewicza 21, Wrocław, Poland; 2 Department of Zoology, Faculty of Natural Sciences, Comenius University, Ilkovičova 6, Bratislava, Slovakia

**Keywords:** Cell growth, Oogenesis, Embryogenesis, Self-renewal

## Abstract

Pseudoscorpions are small matrotrophic chelicerates. The embryos develop in a brood sac and feed on the nutritive fluid provided by the female. It was widely accepted that the nutritive fluid is synthesized in the ovary. Recent studies have shown that in *Chelifer cancroides*, a representative of Cheliferidae, considered one of the most derived pseudoscorpion families, the nutritive fluid is produced not only in the ovary but also in the oviducts. Since evolution of adaptations for matrotrophy in pseudoscorpions is poorly known, we aimed to verify our hypothesis that pseudoscorpions of the family Chernetidae, closely related to Cheliferidae, share the traits of adaptations to matrotrophy in the structure and function of the female reproductive system with *C. cancroides*. We analysed the structure of the ovary and oviducts in five representatives of chernetids with light, confocal, and transmission electron microscopy. The results confirmed our hypothesis and provided new data which broaden our knowledge of matrotrophic pseudoscorpions. We show that in chernetids, the ovary and oviducts undergo significant alterations including their size, multistep hypertrophy and polyploidization of the epithelial cells involved in secretion of the nutritive fluid, the complex secretory activity of the epithelial cells, massive degeneration of the epithelial cells that have completed secretion, and epithelium renewal.

## Introduction

In animals, developing embryos are supported with nutrients deposited during oogenesis in the cytoplasm of oocytes or provided by a maternal organism during embryonic development. The former mode is defined as lecithotrophy, and the latter as matrotrophy, sometimes also referred to as extraembryonic nutrition^1^.

Matrotrophic development is widespread in the animal kingdom, both among invertebrates and vertebrates, including placental mammals. It has evolved independently over a hundred times in different taxa^1^. During evolution, variable patterns of matrotrophic development have developed. As a consequence, matrotrophic animals differ in the amount of nutrients provided by maternal organisms in relation to overall provisioning, from an insignificant amount (incipient matrotrophy) to a substantial one (substantial matrotrophy)^2–5^. Another difference concerns the source of the nutrients. On the basis of the source of nutrients, five main types of matrotrophy have been distinguished: (i) histotrophy, (ii) histophagy; (iii) oophagy, (iv) embryophagy, and (v) placentotrophy. Placentotrophy and histotrophy are the most common, and although placentotrophy is considered the most advanced type of matrotrophic development, histotrophy is the simplest^1^.

In arthropods lecithotrophy predominates, however, the occurrence of extraembryonic nutrition has been evidenced in both subphyla, that is, Mandibulata (Myriapoda and Pancrustacea) and Chelicerata. In the former group, matrotrophy occurs in several insect orders, e.g., Dermaptera, Strepsiptera, Diptera, and some crustaceans e.g., Isopoda and Decapoda^1,6–9^. In extant chelicerates, matrotrophy has been described in pseudoscorpions and scorpions, and 2 genera of mites^10,11^. What is surprising, recent studies on a representative of Pycnogonida, a sister branch of Euchelicerata, indicate that the switch to lecithotrophy happened in recent evolutionary history^12^.

In pseudoscorpions and scorpions, matrotrophy is considered an obligatory mode of embryo feeding^1^. Phylogenetic relationships between pseudoscorpions and scorpions, as well as their position among other chelicerate orders, remain controversial (see e.g.,^13,14^). The latest analyses support the inclusion of pseudoscorpions in Arachnopulmonata as the sister group of scorpions. A new clade named Panscorpiones has been proposed that unites Scorpiones and Pseudoscorpiones^15^.

Scorpiones and Pseudoscorpiones, as matrotrophic chelicerates, have drawn the attention of developmental biologists^16–21^. Comparative analyses clearly indicate that scorpions and pseudoscorpions display a different (taxon-specific) set of characters concerning matrotrophic development and hence it is assumed that in these taxa, matrotrophy has evolved independently^22^.

Scorpions include some of the largest chelicerates. The embryos develop inside the female body, in the ovary, which is composed of an extensive network of longitudinal and transverse tubules. Due to the dual role of the female gonad, it is usually termed the ovariuterus. In basal scorpion families, referred to as apoikogenic, the embryos develop in the lumen of the ovariuterine tubules and feed mainly on reserve materials deposited in the ooplasm. However, this feeding is supported by nutrients that pass from hemolymph through the wall of the ovariuterine tubules. In advanced scorpions, termed katoikogenic, embryonic development occurs in diverticula, which are blind outpocketings of the ovariuterine tubules, and is based on the transfer of nutrients from the hepatopancreas via an elongated appendix, the special organ that develops at the apex of each diverticulum prior to the commencement of embryogenesis^23^.

Pseudoscorpions are small chelicerates. The embryos develop outside the mother’s body, in a brood sac located under the opisthosoma^16,24^. Pseudoscorpions usually accumulate in the ooplasm a small amount of proteid yolk and a great amount of lipids^16,25^. It is widely accepted that developing embryos are fed on the nutritive fluid produced in the female gonads^16,24^. To absorb the nutritive fluid, the embryos develop a unique and transient structure called a pumping organ^16^. Involvement of the somatic cells of the female reproductive system in production of the nutritive fluid is an adaptation for matrotrophic development in pseudoscorpions.

Pseudoscorpion female gonads, like in other small sized arachnids, are reduced to a single tubule^26,27^. The structure of the ovary is typical of chelicerates and classified as the Chelicerate-type^28,29^. The youngest germline cells (oogonia and early meiotic oocytes) are distributed along the long axis of the ovarian wall, which is also referred to as a germarium. With the beginning of previtellogenesis, the first stage of oocyte growth, when organelles and macromolecules accumulate in the cytoplasm of the oocyte, the oocytes expand and protrude from the ovarian wall to the surface of the ovary. In pseudoscorpions, unlike most chelicerates, the oocytes are enclosed by follicular cells that originate from interstitial cells located in the germarium. Bulged to the body cavity the oocytes continue previtellogenic growth and enter vitellogenesis during which they gather reserve materials^30,31^. The connection between the oocytes exposed to the body cavity and the ovarian wall is maintained by the oocyte stalks formed by the epithelial cells that stem from the ovarian epithelium. The oocytes grow asynchronously, and only a part of them, which are the most advanced in development, becomes ovulated.

What is significant for pseudoscorpions, but different from non-matrotrophic chelicerates, following ovulation, the oocyte growth ceases for a period of the embryo development and nutrition. Thus, the ovary functions in two phases (i) oogenic, when the oocyte growth occurs, and (ii) secretory (functional), when the nutritive fluid for developing embryos is produced^19^.

Pseudoscorpiones is a diverse and ancient order of arachnids comprising 26 extant families belonging to nine superfamilies. Cheliferoidea is considered the most derived superfamily of pseudoscorpions and includes four families (i) Withiidae, (ii) Atemnidae, (iii) Chernetidae, and (iv) Cheliferidae. The structure of the female reproductive system of cheliferoids is poorly known except for *Chelifer cancroides* (Linnaeus, 1758)*,* a representative of Cheliferidae. Cheliferidae is among the most derived pseudoscorpion families^13^. The embryonic development of *C. cancroides* is considered to show the most specialized traits^16^. Our previous investigations^32^ revealed that in *C. cancroides*, secretion of the nutritive fluid is amplified due to the involvement in this process not only the epithelial cells of the ovary but also the oviducts. We also showed that the somatic cells that participate in the synthesis of nutritive fluid become highly enlarged as a result of multistep hypertrophy and polyploidization. Our detailed analyses revealed that secretion of the nutritive material starts much earlier than previously reported^16^. Unfortunately, due to our limited knowledge of pseudoscorpion structure of the female reproductive system, it still remains unanswered whether aforementioned characters are unique for Cheliferidae or are common traits of the families closely related to Cheliferidae or even the entire superfamily Cheliferoidea.

The aim of the study was to answer the question if representatives of Chernetidae show similar structure of the female reproductive system to *C. cancroides*. We hypothesized that in chernetids, the secretory phase of the ovarian cycle strongly resembles that described in *C. cancroides*. To address this question and verify our hypothesis, we analyzed the structure of the ovaries and oviducts during the oogenic and secretory phases of the ovarian cycle in five chernetid species, *Allochernes wideri, Chernes hahnii*, *Lamprochernes* sp*., Pselaphochernes lacertosus* and *Pselaphochernes scorpioides* and by means of light, confocal, and transmission electron microscopy.

## Results

### Gross morphology of the female reproductive system

The results presented below pertain to all species unless some differences are indicated.

**Figure 1:**
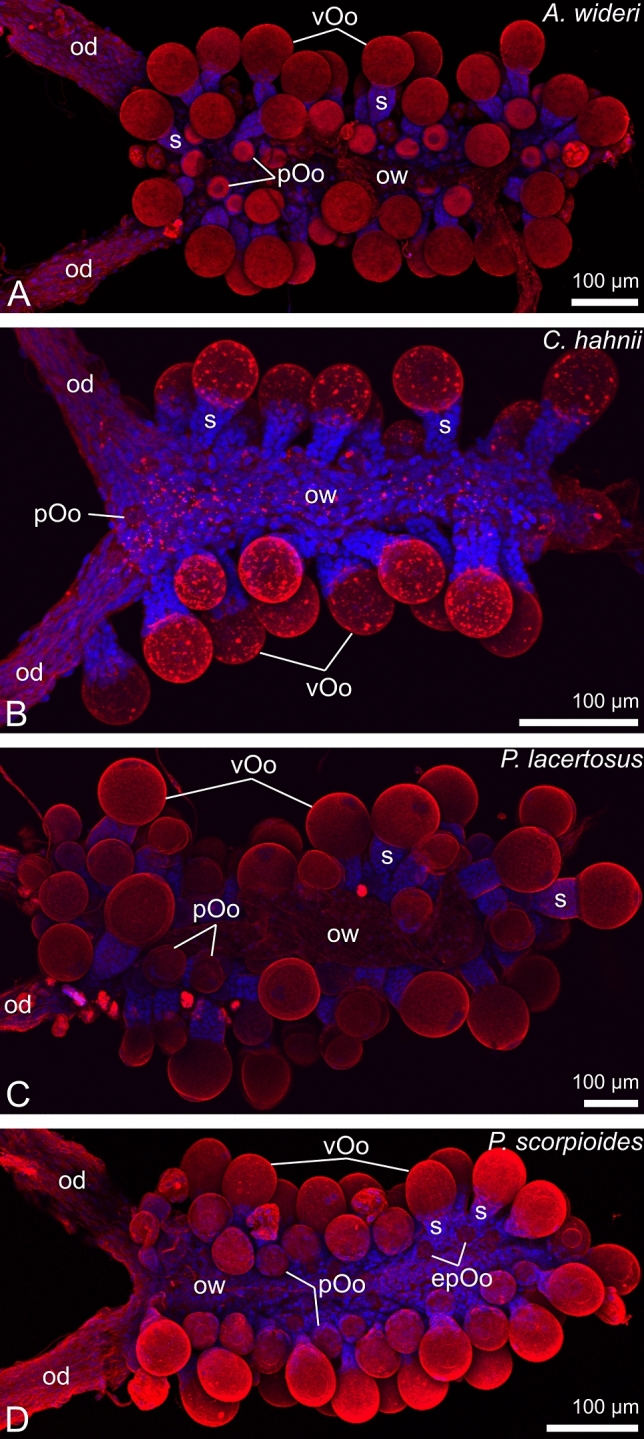
Morphology of the ovaries and oviducts in oogenic phase of the ovarian cycle in *Allochernes wideri* (**A**), *Chernes hahnii* (**B**), *Pselaphochernes lacertosus* (**C**) and *Pselaphochernes scorpioides* (**D**). (**A**–**D**) Each ovary consists of the ovarian wall and oocytes at different stages of previtellogenic and vitellogenic growth located on the ovary surface. Anteriorly ovaries are connected with paired oviducts. In (**B**) note the oocyte located on the surface of the oviduct. (**A**–**D**) Confocal image of wholemount preparation stained with DAPI (4ʹ,6diamidino-2phenylindole dihydrochloride) (blue fluorescence), and Texas-Red-X Agglutinin (red fluorescence). epOo, early previtellogenic oocyte; od, oviduct; ow, ovarian wall; pOo, previtellogenic oocyte; s, stalk; vOo, vitellogenic oocyte.

**Figure 2:**
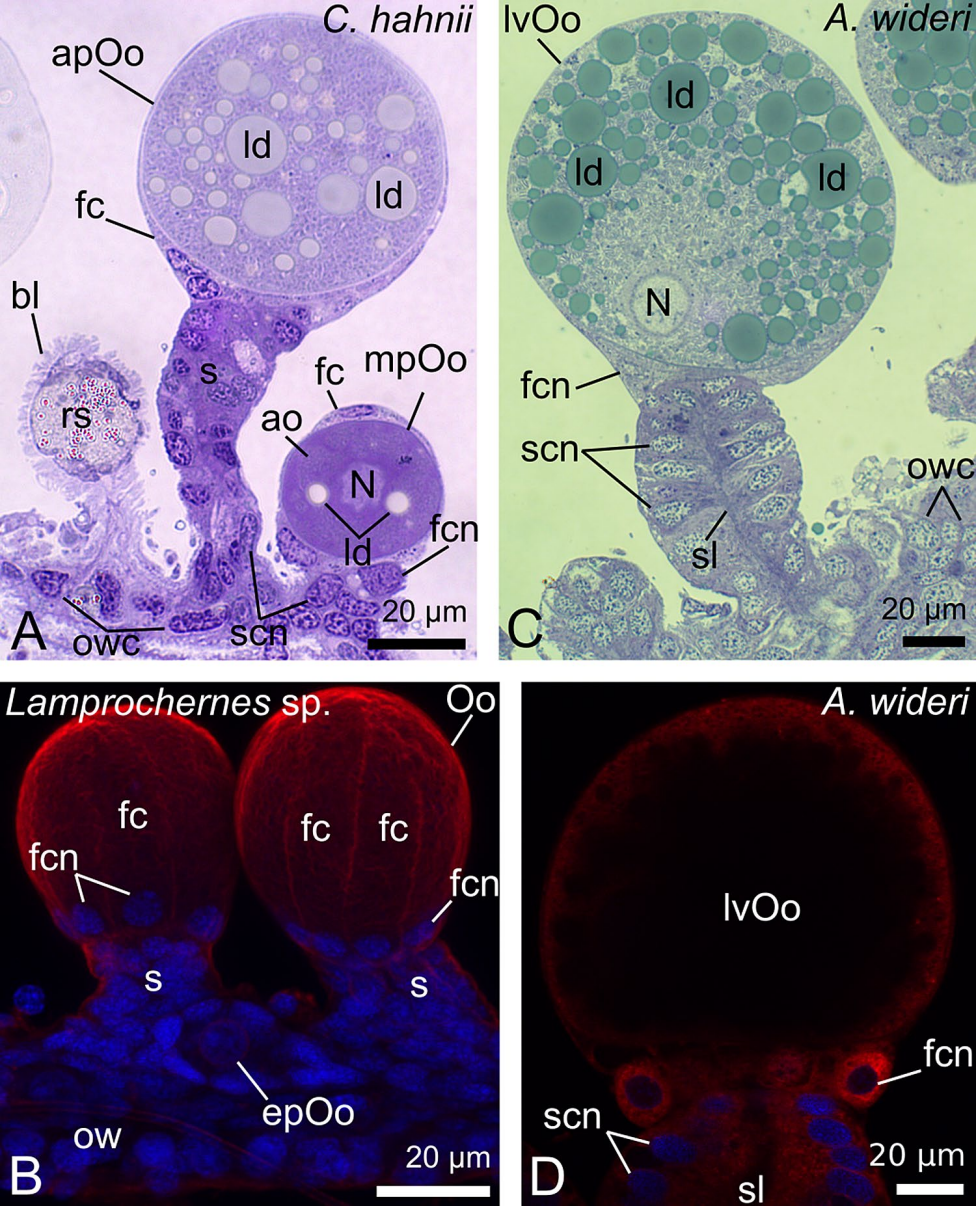
Morphology of the ovary in different stages of the oogenic phase in *Chernes hahnii* (**A**), *Lamprochernes* sp. (**B**) and *Allochernes wideri* (**C**,**D**). (**A**,**B**) In the early stages of the oogenic phase, the early previtellogenic oocytes are located in the ovarian wall, and mid- and advanced previtellogenic oocytes protrude on the ovary surface. In mid-previtellogenesis, the ooplasm contains aggregates of organelles and first lipid droplets, while in advanced previtellogenesis, the voluminous lipid droplets predominate. With the progress of previtellogenic growth, the oocyte stalks become longer due to a growing number of the stalk cells, and the follicular cells become arranged meridional on the oocytes surface with nuclei located near the oocyte proximal pole. The stalk cells and the ovarian wall cells are small and alike. The regressed postovulatory stalks located on the ovary surface indicate that the female undergoes the next round of the ovarian cycle. (**C**,**D**) In the late stages of the oogenic phase, a part of the oocytes bulged on the ovary surface undergoes vitellogenesis. The oocyte nucleus of vitellogenic oocyte is shifted to the peripheral part. The ooplasm is filled with numerous and voluminous lipid droplets and small number of tiny yolk spheres. Stalk cells and epithelial cells of the ovarian wall are hypertrophic and polyploid. The stalk gains the lumen. The cytoplasm of the stalk cells and follicular cells show a positive signal for ER-Tracker staining (**D**). (**A**,**C**) Semi-thin sections stained with methylene blue. (**B**) Confocal image of wholemount preparation stained with DAPI (4ʹ,6diamidino-2phenylindole dihydrochloride) (blue fluorescence), and Texas-Red-X Agglutinin (red fluorescence). (**D**) Confocal image of wholemount preparation stained with DAPI (4ʹ,6diamidino-2phenylindole dihydrochloride) (blue fluorescence), and ER Tracker Red (red fluorescence). ao, aggregates of organelles; apOo, advanced previtellogenic oocyte; bl, basal lamina; epOo, early previtellogenic oocyte; fc, follicular cell; fcn, follicular cell nucleus; ld, lipid droplet; lvOo, late vitellogenic oocyte; mpOo, mid-previtellogenic oocyte; N, oocyte nucleus; Oo, oocyte; ow, ovarian wall; owc, ovarian wall cell; rs, regressed postovulatory stalks; s, stalk; scn, stalk cell nucleus; sl, stalk lumen.

**Figure 3:**
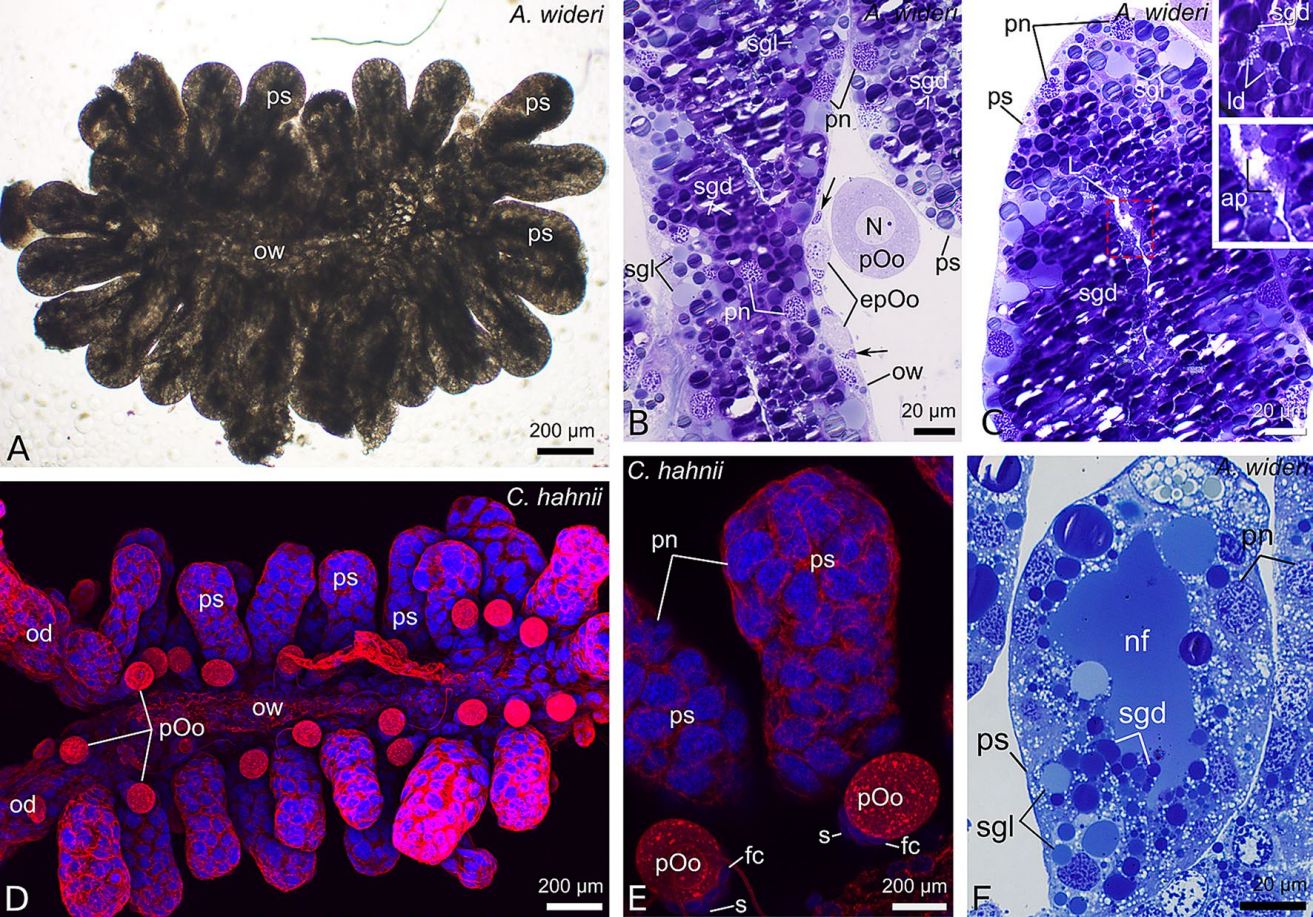
Morphology of the ovary and oviducts in the early stage of the secretory phase (following ovulation) in *Allochernes wideri* (**A**–**C**,**F**) and *Chernes hahnii* (**D**,**E**). (**A**–**F**) In the secretory phase the ovary and oviducts (**A**,**B**) considerably increase in size. In the ovary, the postovulatory stalks predominate (**A**,**D**), which became several times bigger than the stalks of the oocytes stopped in previtellogenesis (**D**,**E**). The postovulatory stalk cells and the epithelial cells of the ovarian wall and the oviducts are highly hypertrophic and polyploid. The cytoplasm of the postovulatory stalk cells and the epithelial cells of the ovarian wall is tightly packed with secretory granules and lipid droplets (**B**,**C**, and upper inset). The early previtellogenic oocytes are located on the surface of the hypertrophic epithelial cells of the ovarian wall. In their closest vicinity, small (non-polyploid) somatic cells are denoted by arrows (**B**). The apical parts of the postovulatory stalk cells start to be detached and enter the lumen of the stalk (**C**, lower inset). In the next stage (**F**) the stalk cells of the postovulatory stalk are lower and have secreted a part of secretory granules. Their lumen is filled with the nutritive fluid which stains light blue. (**A**) Whole mount preparation viewed in Nomarsky optics. (**B**,**C**,**F**) Semi-thin sections stained with methylene blue. (**D**,**E**) Confocal image of wholemount preparation stained with DAPI (4ʹ,6diamidino-2phenylindole dihydrochloride) (blue fluorescence), and Texas-Red-X Agglutinin (red fluorescence). ap, apical part of epithelial cell; epOo, early previtellogenic oocyte; fc, follicular cell; L, lumen; ld, lipid droplet; N, oocyte nucleus; nf, nutritive fluid; od, oviduct; ow, ovarian wall; pn, polyploid nucleus; pOo, previtellogenic oocyte; ps, post-ovulatory stalk; s, stalk; sgd, dark secretory granules; sgl, light secretory granules.

**Figure 4:**
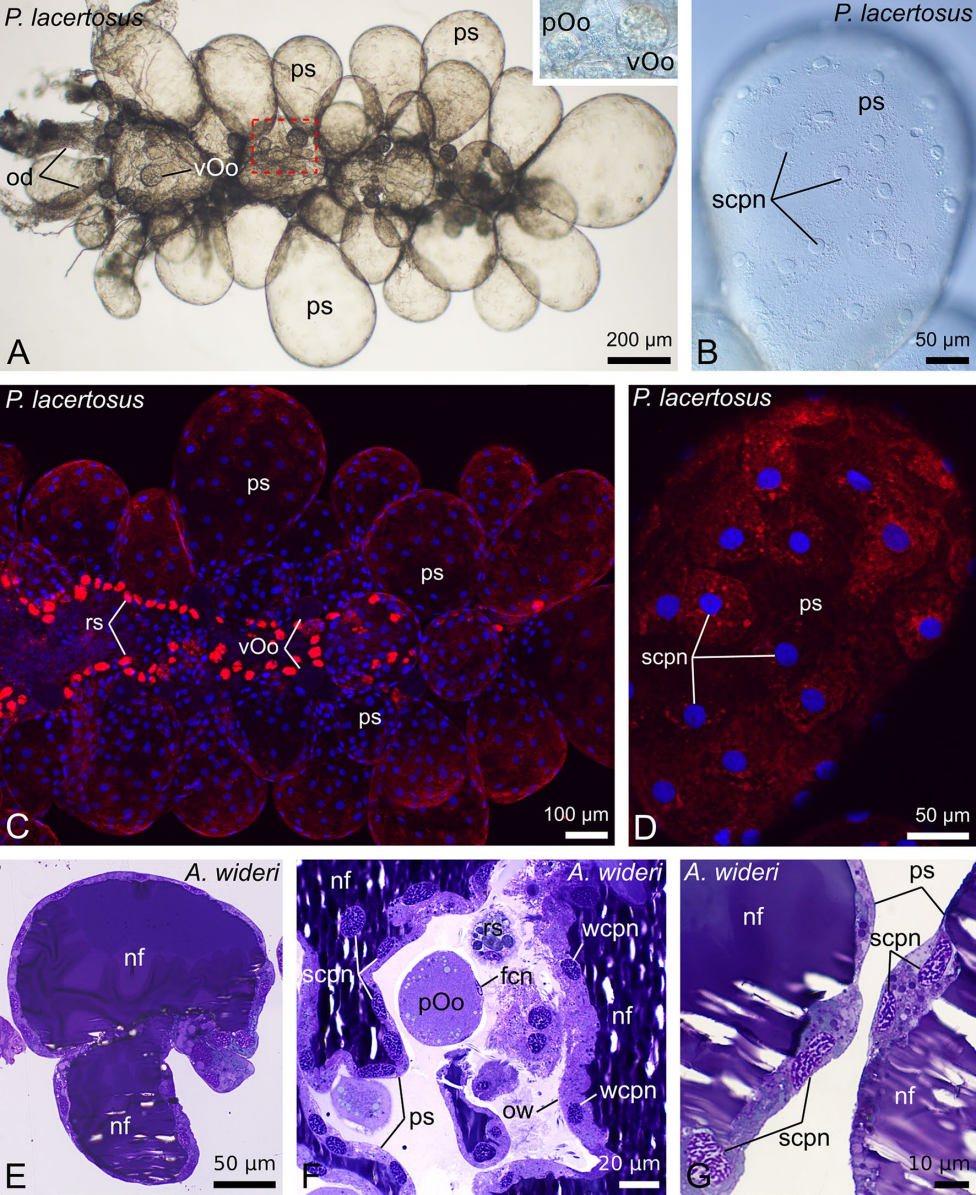
Morphology of the ovary and oviducts in the late secretory phase in *Pselaphochernes lacertosus* (**A**–**D**) and *Allochernes wideri* (**E**–**G**). (**A**–**G**) During advanced secretory phase the volume of the gonad and the oviducts is increased. The oocytes stopped in previtellogenic or early vitellogenic growth are located close to the ovarian wall among the regressed postovulatory stalks that remained after the previous secretory phase (**A**, and inset, **C**). The postovulatory stalks are swollen and voluminous (**A**,**C**). The cells of postovulatory stalks and epithelial cells of the ovarian wall are flat with flattened polyploid nuclei (**B**,**D**,**F**,**G**). The lumen of the latter stalks and the lumen of the ovary is filled with the nutritive fluid which stains dark blue. (**A**,**B**) Whole mount preparation viewed in Nomarsky optics. (**C**,**D**) Confocal image of wholemount preparation stained with DAPI (4ʹ,6diamidino-2phenylindole dihydrochloride) (blue fluorescence), and Texas-Red-X Agglutinin (red fluorescence). (**E**–**G**) Semi-thin sections stained with methylene blue. fcn, follicular cell nucleus; nf, nutritive fluid; od, oviduct; ow, ovarian wall; pOo, previtellogenic oocyte; ps, postovulatory stalk; rs, regressed postovulatory stalks; scpn, stalk cell polyploid nucleus; vOo, vitellogenic oocyte; wcpn, ovarian wall polyploid cell nucleus.

**Figure 5:**
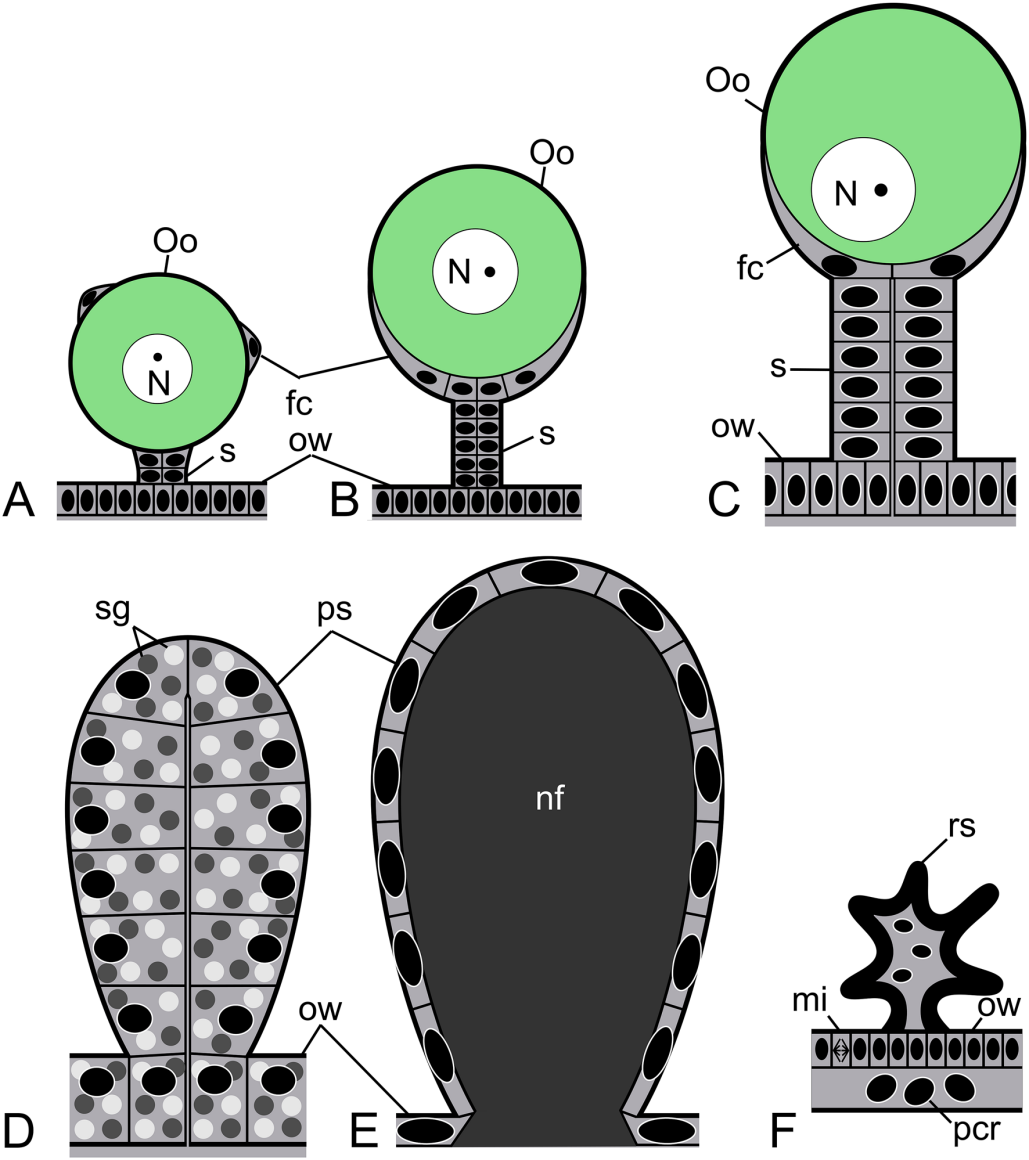
Diagram illustrating changes in the morphology of the stalks and the ovarian epithelium before (**A**–**C**) and after (**D**–**F**) ovulation (not to scale). For simplicity, the epithelium of the oviduct, which undergoes similar changes to the ovarian epithelium, and the musculature of the ovary and oviducts are not shown. (**A**) Early oogenic phase. Early previtellogenic oocyte bulges from the ovarian wall covered by follicular cells. The stalk consists of a few cells. (**B**) Advanced oogenic phase. Advanced previtellogenic/early vitellogenic oocyte is surrounded by follicular cells whose nuclei are located at the oocyte proximal pole. The oocyte stalk is built of several roughly cuboidal cells similar to the epithelial wall cells. (**C**) Late oogenic phase. Late vitellogenic oocyte is exposed to the body cavity. The oocyte stalk and the ovarian epithelium consist of hypertrophic columnar and polyploid cells. (**D**) Early secretory phase. The postovulatory stalk and the ovarian epithelium consist of highly hypertrophic and polyploid cells whose cytoplasm is occupied by numerous secretory granules. (**E**) Advanced secretory phase. The wall of the postovulatory stalk and the ovarian epithelium is built of the polyploid cells that have secreted most of their cytoplasmic content to the lumen of the stalk and the ovary. The nutritive fluid fills the lumen of the stalk and the ovary. (**F**) Late secretory (regeneration) phase. After secretion of the nutritive fluid, the polyploid epithelial cells of the ovarian wall degenerate and delaminate from the epithelium. The ovarian epithelium is replaced by mitotically active non-polyploid epithelial cells. The postovulatory stalk shrinks and remains on the ovary surface during consecutive ovarian cycles as the regressed postovulatory stalk. fc, follicular cell; mi, mitotic cell division; N, oocyte nucleus; nf, nutritive fluid; Oo, oocyte; ow, ovarian wall; pcr, polyploid cell remnants; ps, post-ovulatory stalk; rs, regressed postovulatory stalk; s, stalk; sg, secretory granules.

**Figure 6:**
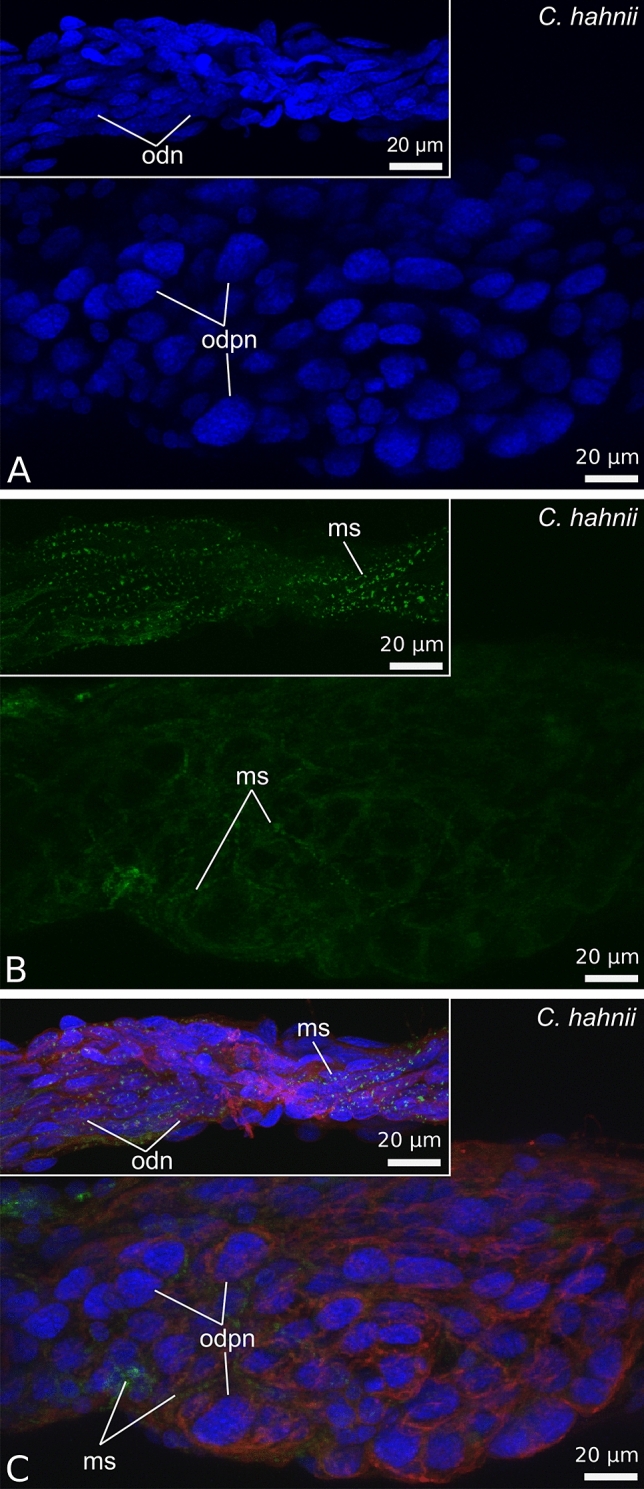
Morphology of the oviducts of *Chernes hahnii* during secretory stage (**A**–**C**) and oogenic stage (insets). (**A**–**C**) In oogenic stage of the ovarian cycle the wall of the oviduct consists of small and elongated epithelial cells covered by a tight network of striated muscles arranged longitudinally. In the secretory phase the epithelial cells are considerably bigger, with large polyploid nuclei. The external muscle cover is clearly extended. (**A**–**C**) Confocal image of wholemount preparation stained with DAPI (4ʹ,6diamidino-2phenylindole dihydrochloride) (blue fluorescence), Alexa Fluor 488 Phalloidin (green fluorescence) and Texas-Red-X Agglutinin (red fluorescence). ms, muscle fibre; odn, oviduct cell nucleus; odpn, oviduct cell polyploid nucleus.

The ovaries of the chernetids analyzed have a typical structure of pseudoscorpions. They are unpaired grape-shaped organs that anteriorly continue into paired oviducts (Fig. 1A–D). The early meiotic oocytes (not shown), and the oocytes that start previtellogenic growth occupy the internal part of the ovarian wall (Fig. 2C). Oocytes at different stages of previtellogenic and vitellogenic growth protrude from the ovarian wall to a body cavity and remain attached to the ovarian tube by means of epithelial stalk cells (Figs. 1A–D and 2A–D). Each protruded oocyte is surrounded by follicular cells (Figs. 2A–D, 3E, 4F and 5A–C). The wall of the ovarian tube and the oviducts is composed of epithelial cells and striated muscles (Figs. 2A–C and 6A–C).

**Figure 7:**
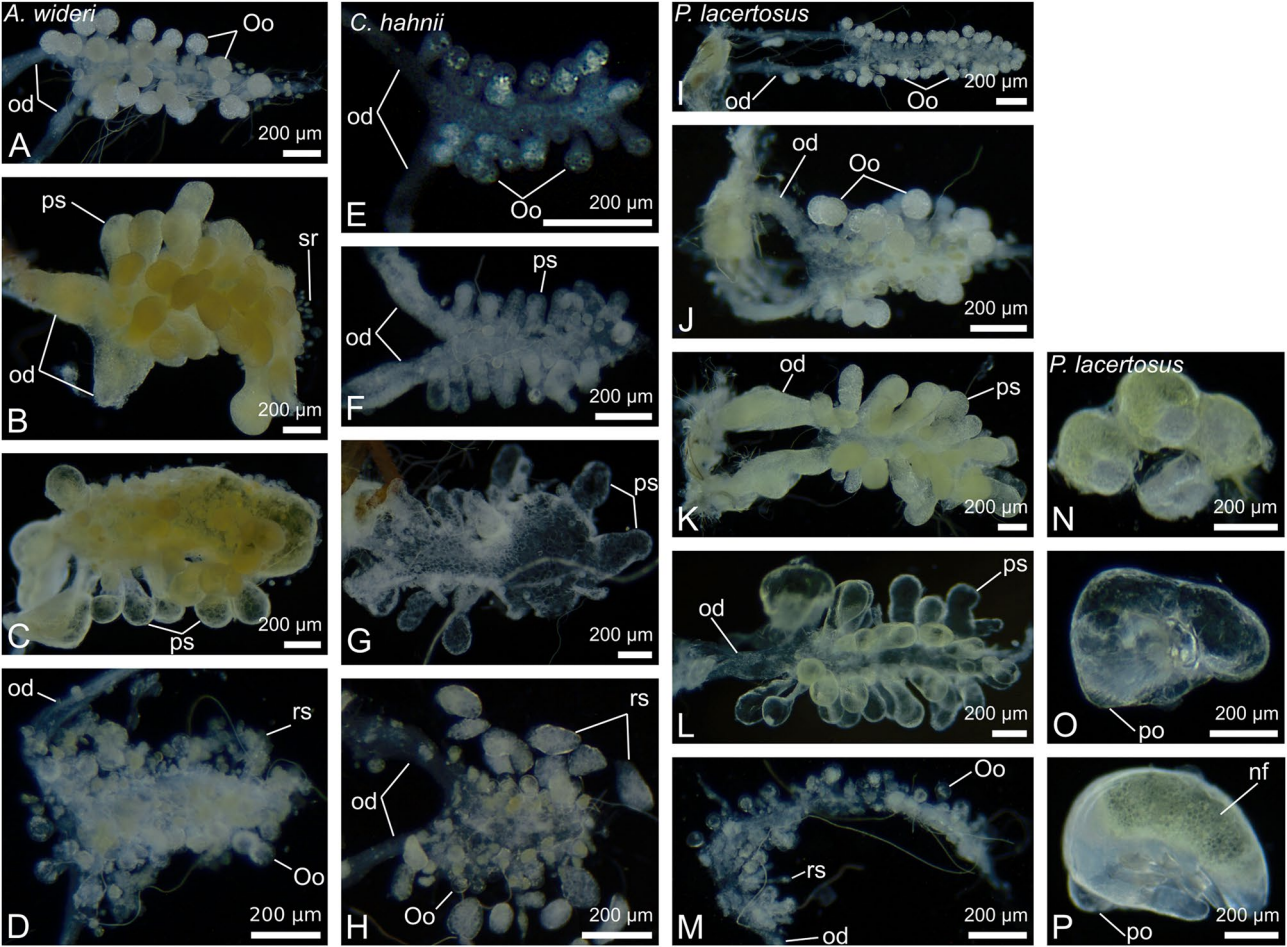
Summarized changes of the morphology of the ovaries and oviducts in *Allochernes wideri* (**A**–**D**), *Chernes hahnii* (**E**–**H**), and *Pselaphochernes lacertosus* (**I**–**M**) during consecutive stages of the ovarian cycle. Photos (**A**,**E**,**I**,**J**) show ovaries in oogenic stage, photos (**B**,**C**,**F**,**G**,**K**,**L**) in the secretory stage, photos (**D**,**H**,**M**) during regeneration after the secretory stage. Pairs of photos (**K**,**N**), (**L**,**O**), (**M**,**P**) show ovaries and embryos in the corresponding stage. (**A**–**P**) Dark-field microscope. nf, nutritive fluid; od, oviduct; Oo, oocyte; po, pumping organ; ps, postovulatory stalk; rs, regressed postovulatory stalk.

Although the general structure of the ovaries in the species is similar, there are also some notable differences. First, the number of oocytes in the most advanced stages of oogenesis, corresponding to the number of laid eggs, varies from circa 20 in *Chernes hahnii* (Fig. 1B) and *Lamprochernes* sp*.* (not shown)*,* 30 in *Allochernes wideri* (Fig. 1A), to 40 in *Pselaphochernes scorpioides* (Fig. 1D) and *Pselaphochernes lacertosus* (Fig. 1C)*.* Second, in some cases, the ovary is anteriorly bifurcated. In *Pselaphochernes lacertosus* (Fig. 7I), the anterior branches show a typical ovary structure and contain a similar number of growing oocytes compared to the unbranched part. In *Chernes hahnii* (Figs. 1B and 7F) and *Allochernes wideri* (Fig. 7A), the anterior bifurcation is not so evident since only single oocytes or post-ovulatory stalks are occasionally observed on the surface of regions that correspond to the oviducts or ovary branches.

### Early and mid oogenic stages

During the early and mid-stages of the oogenic phase, the oocytes that have bulged on the ovary surface undergo previtellogenic growth. The volume of the oocytes is relatively small. The germinal vesicles occupy the cell centre (Fig. 2A), and the oocytes cytoplasm (ooplasm) is filled with a growing number of organelles. The organelles gather in groups that are clearly visible in the light microscopy. Among the accumulated organelles first lipid droplets appear. Follicular cells are unevenly distributed on the surface of growing oocytes. The oocyte stalks are built of a few relatively small roughly cuboidal cells, grouped at the proximal pole (close to the ovarian wall) of the oocytes (Figs. 2A and 5A).

**Figure 8:**
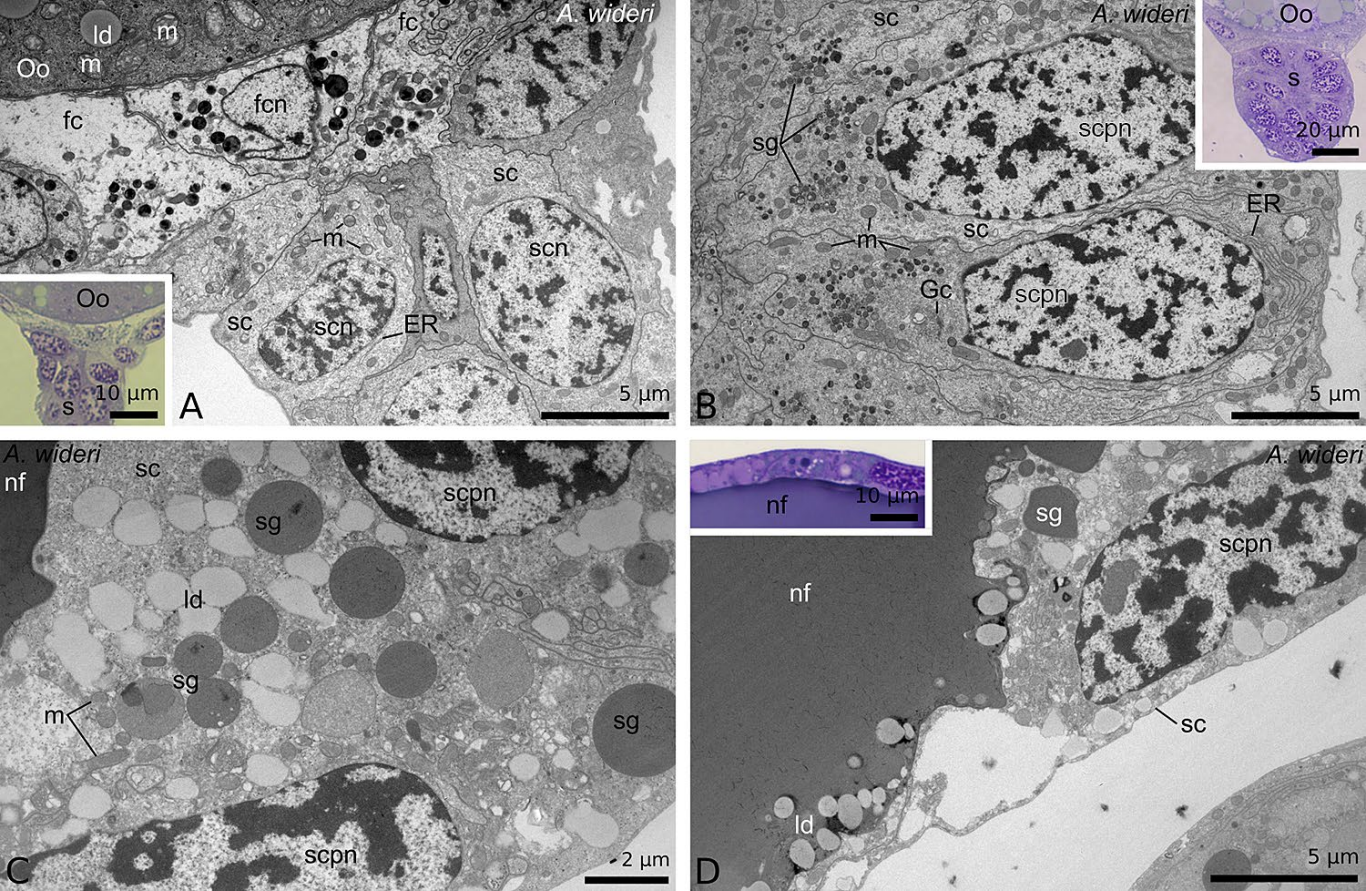
Ultrastructure of the preovulatory (**A**,**B**) and postovulatory (**C**,**D**) stalk cells in *Allochernes wideri*. (**A**) In the early oogenic phase the stalk cells which form the stalk of previtellogenic oocyte are cuboidal with small number of organelles. (**B**) In advances oogenic phase the stalk of the early vitellogenic oocyte is composed of columnar stalk cells with numerous organelles including endoplasmic reticulum, mitochondria, Golgi complexes, and small secretory vesicles. The nuclei of stalk cells are enlarged. (**C**,**D**) In the late secretory phase the stalk cells have a reduced hight. Their cytoplasm contains a small number of secretory granules and lipid droplets. The polyploid nuclei are elongated and oriented longitudinal to the basal pole. The lumen of the stalks is filled with nutritive fluid of moderate or electron-dense structure. In the fluid lipid droplets are noticeable close to the apical parts of the stalk cells. (**A**–**D**) TEM. (**A**,**B**,**D** insets) Semi-thin sections stained with methylene blue. ER, endoplasmic reticulum; fc, follicular cell; fcn, follicular cell nucleus; Gc, Golgi complexes; ld, lipid droplet; m, mitochondria; nf, nutritive fluid; Oo, oocyte; s, stalk; sc, stalk cell; scn, stalk cell nucleus; scpn, stalk cell polyploid nucleus; sg, secretory granules.

In advanced previtellogenesis, the size of the oocytes increases, and the ooplasm contains numerous and large lipid droplets (Fig. 2A). Elongated follicular cells are arranged meridional (from proximal to distal pole of the oocyte) on the oocytes surface, and their nuclei are shifted at the proximal pole of the oocyte, near the stalk cells (Fig. 2B). The oocyte stalks consist of a higher number of cells and become longer (Figs. 2A and 5B). The size of the stalk cells is comparable to that of the stalk cells of younger oocytes. The nuclei of stalk cells are located at the cell centre, and their cytoplasm is filled with a small number of organelles, where ER elements and mitochondria predominate. There is no lumen in the stalk at this stage (Figs. 2A and 8A).

The epithelium of the ovarian tube is simple cuboidal (Fig. 2A). The wall of the oviducts is composed of low epithelial cells with elongated nuclei aligned along a long axis of the oviduct (Fig. 6A–C insets).

### Late oogenic stage (before ovulation)

Close to ovulation, the oocytes enlarge during vitellogenic growth. The ooplasm is loaded with a significant number of lipid droplets, and most of them have a great diameter. Among the lipid droplets, very few small dark stained granules, the yolk spheres, are distributed (Fig. 2C). The germinal vesicle is shifted to the peripheral ooplasm (Fig. 2C), which suggests that the oocytes are close to reaching their maturity and in a short time would be ready for ovulation. The stalk cells of vitellogenic oocytes, epithelial cells of the ovary and the oviducts became hypertrophic and polyploid (Figs. 2C, 6A–C and 5C). As a consequence, the shape and size of cells change from small cuboidal to high columnar (Fig. 2C,D). The cytoplasm of pre-ovulatory stalk cells is rich in organelles such as ER cisterns, Golgi complexes, mitochondria, and small secretory vesicles (Figs. 2D and 8B). The secretory vesicles gather in the apical part of the cell, and the cytoplasm of the basal part is occupied by large polyploid and elongated nuclei (Fig. 8B). The stalks of preovulatory oocytes are long and wide with a length comparable to the diameter of vitellogenic oocytes. In the stalks, a narrow lumen appears and the stalks become open (Figs. 2C,D and 5C).

### Early secretory phase (after ovulation)

After ovulation, which is based on the passage of mature oocytes through the lumen of their stalks, the structure of the ovary and oviducts significantly changes. Their size considerably increases compared to the preovulatory stage (Fig. 7A,B,E,F,J,K). The postovulatory stalks become the most voluminous (Figs. 3A,C,D,E, 7B,F,K and 5D). The length of the postovulatory stalks is many times larger compared to the oogenic phase, which is clearly noticeable when the size of the postovulatory stalks is compared to the stalks of the oocytes arrested in early previtellogenesis (Fig. 3D,E). The morphology of the postovulatory stalk cells, the epithelial cells of the ovarian wall, and the oviduct is alike. They continue hypertrophic growth and polyploidization, and eventually become huge and highly columnar (Figs. 3B,C and 5D). Their polyploid nuclei reach the largest size and maintain the basal position, while their cytoplasm is densely filled with granules (Figs. 3B,C and 5D). The granules are large and represent two categories. One of them, which predominate in the cytoplasm, are intensely stained with methylene blue, whereas, the other, less numerous, show weaker coloration (Fig. 3B,C). In this study, the content of heterogeneous granules was not analyzed with histochemical methods. Among the granules, small lipid droplets are distributed (Fig. 3C upper inset). Hypertrophic epithelial cells start releasing their content to the lumina of the ovary and postovulatory stalks, and the oviduct (not shown) by detaching small fragments from their apical parts (Fig. 3C lower inset). At this stage of the ovarian cycle, the embryos located in the brood sac are in the early stages of development, that is, before the development of the pumping organ (Fig. 7N).

The youngest oocytes in the early meiotic stages change their position compared to the oogenic stage and are located externally to the hypertrophic and polyploid ovarian epithelial cells. In their close neighbourhood small somatic cells are discernible (Fig. 3B). The latter cells do not show any characteristic of hypertrophy or polyploidy. Oocytes arrested in previtellogenesis remain bulged to the body cavity on their short stalks, so close to the ovarian tube (Fig. 3B,D,E).

### Advanced secretory phase (nutritive fluid secretion)

In the subsequent stage, secretion of the nutritive fluid starts. At first, the fluid appears in the lumen of the postovulatory stalks. The lumen of stalks widens, and the height of the stalk cells becomes reduced, but their cytoplasm still contains numerous secretory granules (Fig. 3F). In the next stage of the secretory phase, the stalks undergo the final increase. They become significantly distended, resembling huge balloons (Figs. 4A–G, 7C,G,L and 5E). The height of epithelial cells that built the wall of the postovulatory stalks, and the ovarian wall becomes considerably decreased, and their lumina become filled with a huge amount of homogenous fluid (Figs. 4E–G and 8D). In the fluid, near the apical membrane of epithelial cells, lipid droplets are observed (Fig. 8D). The polyploid nuclei become elongated and oriented perpendicular to the apical-basal axis of epithelial cells, and the number of secretory granules and lipid droplets is relatively low (Figs. 4B,D,E and 8C,D). At that stage, the embryos located in the brood sac have developed the pumping organ (Fig. 7O), so they are ready to absorb the nutritive fluid.

### Late secretory stage (regeneration)

**Figure 9:**
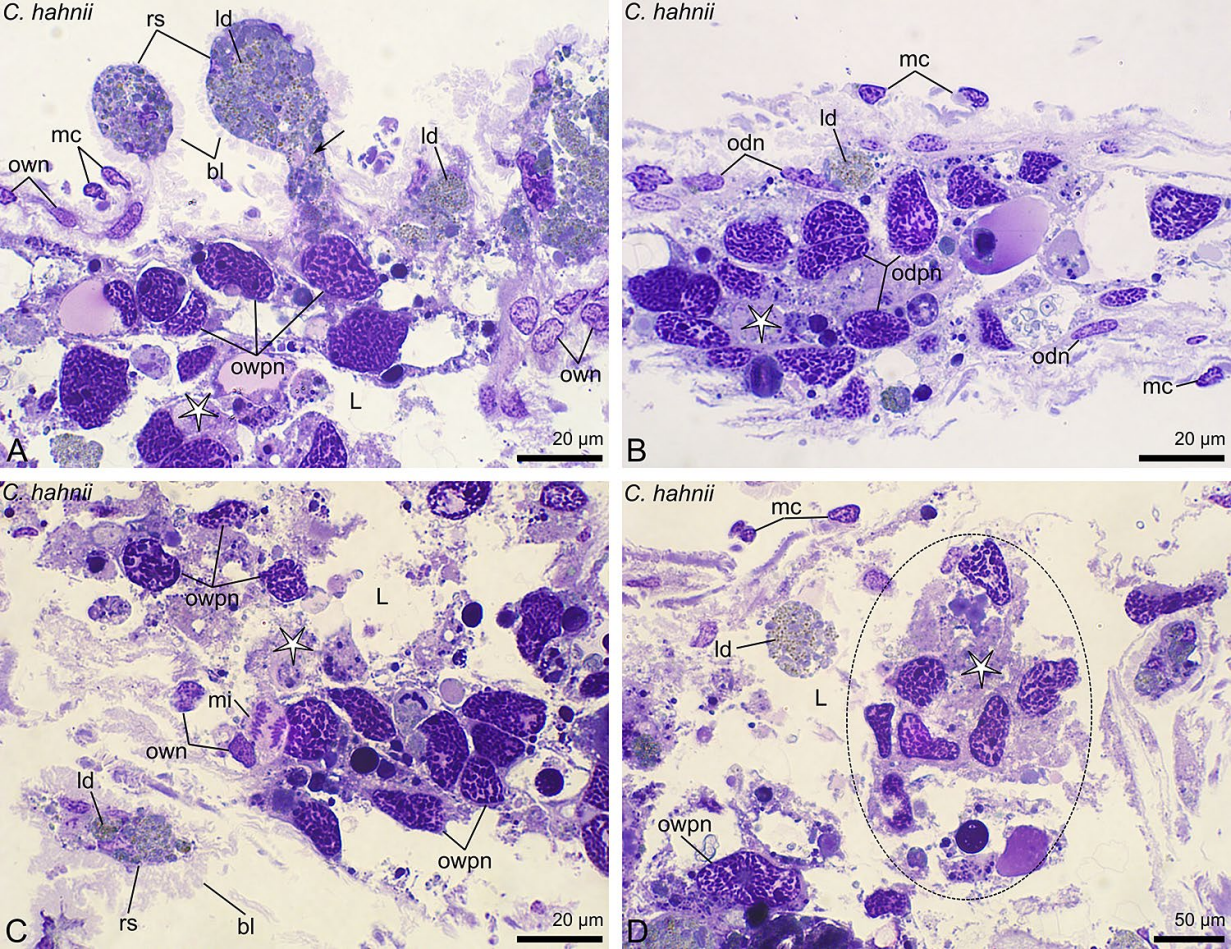
Structure of the ovary (**A**,**C**,**D**) and oviduct (**B**) of *Chernes hahnii* during regeneration after the secretory stage. (**A**–**D**) After completion of secretion of the nutritive fluid, the epithelial cells of the ovarian and oviductal wall degenerate and delaminate from the epithelium. Their polyploid nuclei and remnants of cytoplasm appear in the lumina of the organs (asterisk). Their place is taken by a new population of epithelial cells that do not show traits of hypertrophy and polyploidization. The postovulatory stalks shrink and remain exposed on the surface of the ovary covered by the folded basal lamina. A part of the cytoplasm content of the stalk cells is released to the lumen of the ovary (arrow). Among degenerating cells mitotically dividing cell is visible. (**A**–**D**) Semi-thin sections stained with methylene blue. bl, basal lamina; L, ovarian lumen; ld, lipid dropled; mc, muscle cell; mi, mitotic cell division; odn, oviduct cell nucleus; odpn, oviduct cell polyploid nucleus; own, ovarian wall cell nucleus; owpn, ovarian wall cell polyploid nucleus; rs, regressed postovulatory stalks.

**Figure 10:**
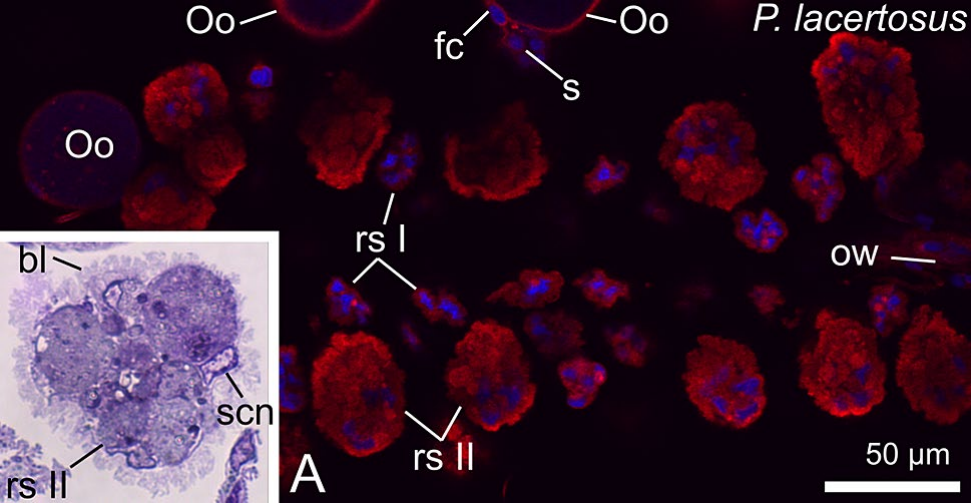
Morphology of the ovary in the third oogenic phase after two ovarian cycles of the *Pselaphochernes lacertosus*. (**A**) The ovary contains previtellogenic oocytes growing on the ovary surface, and two types of regressed postovulatory stalks that differ in the size. The smaller regressed postovulatory stalks are the remnants of the postovulatory stalks after completion of the first ovarian cycle, and the bigger ones represent the remnants of the postovulatory stalks of the second ovarian cycle. Inset: The regressed postovulatory stalks after the second ovarian cycle contain stalk cells covered with the highly folded basal lamina. (**A**) Confocal image of wholemount preparation stained with DAPI (4ʹ,6diamidino-2phenylindole dihydrochloride) (blue fluorescence), and Texas-Red-X Agglutinin (red fluorescence). (inset) Semi-thin section stained with methylene blue. bl, basal lamina; fc, follicular cell; Oo, oocyte; ow, ovarian wall; rs I, regressed postovulatory stalks after first ovarian cycle; rs II, regressed postovulatory stalks after second ovarian cycle; scn, nucleus of the regressed stalk cell; s, stalk.

Once the secretion of the nutritive fluid has been completed and the fluid has left the ovary and the oviduct, the structure of both organs of the female reproductive system changes dramatically and exhibits traits of massive cell degeneration (Fig. 7D,H,M). The embryos developing in the brood sac have already absorbed the nutritive fluid, which is visible inside the embryo's body, in the lumen of a digestive system (Fig. 7P). At this stage, polyploid epithelial cells of the ovarian and oviduct walls degenerate and delaminate from the epithelium. Their cell membranes disintegrate, and polyploid nuclei and remnants of cytoplasm appear in the lumen of the ovary and oviduct (Fig. 9A–D). The place of degenerated cells is taken by a new generation of small non-polyploid epithelial cells. During this time mitotically dividing somatic cells are observed in the ovary (Figs. 9C and 5F) and the oviducts (not shown) which strongly suggests that the epithelium renewal occurs due to mitotic divisions of the somatic cells that have not underwent hypertrophy and polyploidization. The postovulatory stalks shrink and stay attached to the external surface of the ovary wall surrounded by a highly folded basal lamina. The cytoplasm of those stalk cells remains filled with numerous lipid droplets (Fig. 9A,C). During the epithelium regeneration, a part of the stalk cells’ content is released to the lumen of the ovary (Fig. 9A arrow). In consecutive stages of the ovarian cycle, the regressed post-ovulatory stalks diminish and become easily distinguishable from the regressed post-ovulatory stalks of the next ovarian cycle (Fig. 10A and inset).

## Discussion

In all investigated species from four genera of the family Chernetidae, the structure of the ovary is similar and typical of chelicerates with growing oocytes exposed to the body cavity. As in other pseudoscorpions, and different from most chelicerates, growing oocytes are enclosed by follicular cells. Previous studies showed that the follicular cells originate from interstitial cells located in the germarium among the youngest germline cells^31^. In chernetids, the arrangement of follicular cells on the oocyte surface is similar to that described in pseudoscorpions from the family Cheliferidae^31^ and Cheiridiidae^27^. It strengthens our previous hypothesis that this is a common feature of all pseudoscorpions.

In small body-sized arachnids such as schizomids^33^, some mites^34–37^, and pseudoscorpions^16^, the ovary is unpaired. In much bigger chelicerates, such as xiphosurans^28,38^, scorpions^23,39–41^, solifuges^42^, amblypigids^43^, and spiders^44^, the ovary is either branched or paired. Reduction of the number of ovarian tubes is considered one of the adaptations to obtain a small or miniature body size^26^.

Pioneering studies on ovary structure in representatives of Garypidae^19,21^ and Chernetidae^16^ revealed that during the secretory phase of the ovarian cycle, the ovary becomes structurally modified. The oocytes growth stops in early previtellogenesis, the ovarian wall thickens, and columnar epithelial cells of the ovarian wall secrete the nutritive fluid. Our previous investigations^31^ showed that in *C. cancroides,* during the secretory phase, the thickening of the ovarian wall is accompanied by the hypertrophy of the stalk cells that do not degenerate after ovulation. Due to this hypertrophy, the postovulatory stalks become the most voluminous structures in the ovary. Our quite recent findings^32^ revealed that in *Chelifer* two organs of the female reproductive system, that is, the ovary and the oviducts, are involved in the synthesis of the nutritive fluid. We also showed that during the secretory phase of the ovarian cycle, stalk cells together with epithelial wall cells of the ovary and oviducts undergo multistep hypertrophy and polyploidization and show structural similarities of secretory active cells^32^. In this study, we demonstrate that in chernetids the ovary and the oviducts undergo structural alterations during consecutive phases of the ovarian cycle, which strongly resemble those described in *Chelifer*. Those changes include an outstanding increase in the size of the organs of the female reproductive system in the secretory phase of the ovarian cycle caused by high-level hypertrophy and polyploidization of the epithelial cells of the ovarian wall, the stalk cells, and the epithelial cells of the oviducts.

Significantly, the most spectacular growth concerns the oocyte stalks which in the late secretory phase extremely enlarge and look like huge balloons filled with homogenous fluid. So far, the occurrence of such huge stalks has never been reported. There is a possibility, that these structures are unique for some pseudoscorpions including chernetids. On the other hand, the lack of such data in the literature could be explained by the fact that during the secretory phase the pseudoscorpion females occupy their nests, so finding the hidden females is quite difficult. In consequence, this exact phase of the ovarian cycle could be easily omitted for investigations. The latter idea seems to be supported by incomplete investigations conducted by Weygoldt^16,45^ on *Pselaphochernes scorpioides*, which lack a description of the postovulatory stalks*.* A similar case might probably concern our previous analyses on the ovary structure in *C. cancroides*^32^.

Previous comparative studies indicate that pseudoscorpions from different families show differences in embryonic development in the amount of reserve materials deposited in the oocytes, the longevity and time of feeding the embryos, and the rate of nutritive fluid production. For example, representatives of chernetids and cheliferids pump the nutritive fluid very early and rapidly, as soon as the pumping organ is functional, while chthoniids pump late and for a long period of time^16^. Some of those differences seem to be closely correlated with the efficiency of nutritive fluid production. As already mentioned, in chernetids, similar to *C. cancroides*^32^, the epithelial cells that produce the nutritive fluid are numerous and represented by three cell populations: (i) the postovulatory stalk cells, (ii) the epithelial ovarian wall cells, and (iii) the epithelial oviductal cells. Otherwise, all mentioned cells are hypertrophic and highly polyploid. It is worth mentioning that hypertrophy of the secretory cells involved in the synthesis of nutrients is quite common among matrotrophic invertebrates, e.g., bryozoans, kamptozoans, bivalves, synascidians and branchiopod crustaceans^1,46^. Polyploidization of secretory epithelial cells is another factor that increases the efficiency of the nutritive fluid synthesis. In chernetids, the amount of nutrients provided by the female for developing embryos seems to be substantial in relation to the small amount of proteid yolk accumulated in the oocytes during vitellogenesis. The high amount of synthesized nutrients also meets the requirements of embryos that share nutrients transferred to the brood sac. It should be reminded here that in chernetids, like in *Chelifer*, the number of growing oocytes and in consequence developing embryos is quite high and amounts to several dozens (^16^ this study). So, it becomes clear that chernetids in terms of the efficiency of the nutritive fluid production show the same strategy as that known in *Chelifer*^32^.

In pseudoscorpions the time of provisioning the embryos with nutrients is diversified. In chernetids and cheliferids it finishes quite early^16^. Subsequently, the secretion of nutrients is stopped and the next round of the ovarian cycle restarts. Before entering the next stage of the cycle, both organs responsible for the secretion of nutrients undergo additional structural modifications. Although previous studies clearly indicated that the ovary structure is modified during the transition from one ovarian cycle to another it was not evident how does it really happen. Chamberlin in his book^24^ only mentioned about rapid degeneration of the ovary which coincides with the extrusion of the egg mass. From Weygoldt’s^16^ studies on *P. scorpioides,* it was known that the epithelial cells of the ovary after secretion reduce their height. Makioka stated that in *Anchigarypus japonicus* the ovarian epithelial cells that have completed the secretion degenerate^19^. Unfortunately, he neither described epithelium degeneration nor referred to the source of stem cells for epithelium renewal. Our previous study on *Chelifer*^32^, assumed that secretory cells after the secretory phase end with break up, but we were unable to prove this.

In this study, we show that at the end of the secretory phase in the ovary and oviducts of chernetids the vast majority of polyploid epithelial cells involved in secretion degenerate. Degeneration does not include post-ovulatory stalk cells that shrink and remain on the surface of the ovary during consecutive stages of the ovarian cycle, indicating the number of previously ovulated oocytes. The elimination of a large number of epithelial cells seems to be related to their previous polyploidization. Despite that polyploidization of epithelial cells undoubtedly favours the efficiency of nutrient secretion, it also makes, that epithelial cells after fulfilling their secretory role become transiently redundant until the next secretory phase of the ovarian cycle. Before the next oogenic stage, the ovarian and oviductal epithelium is renewed due to mitotic activity of the cells that have remained unchanged by polyploidization and hypertrophy. The best candidates for stem cells in epithelium renewal are the somatic cells that remain on the surface of the ovarian wall in the close vicinity to the early previtellogenic oocytes. These somatic cells are at early stages of differentiation, and their descendants that appear in the ovary follow the same pattern of development as their ancestors and initially differentiate into the stalk cells of oocytes, which restart previtellogenic growth, and rebuild the wall of the ovary and the oviduct. Eventually, in the next secretory phase, they undergo hypertrophy, polyploidization, etc.

To our knowledge, this is the first report showing the renewal of the epithelium in the female reproductive system of pseudoscorpions. It is worth underlying that degeneration of somatic cells and in consequence the epithelium renewal occur on a great scale, so with great metabolic costs. Similar periodic destruction and regeneration of the epithelium in the female reproductive system is well known across the animal kingdom, including human beings and in the primates during menstruation, and in the lower mammals during estrus^47^. It seems probable that in chernetids, the cells remnants of degenerating epithelial cells are not wasted but become transferred to the brood sac in the second round of the nutrients provisioning. It needs to be confirmed in future studies.

Another interesting issue worth considering is the mode of nutritive fluid secretion. The epithelial cells responsible for this process accumulate in their cytoplasm a huge number of secretory granules that exhibit a heterogeneous structure. The process of secretion is quite complex and generally well fits the apocrine mode (see e.g.,^48,49^). Secretion starts with defragmentation of the apical part of the cell, proceeds by releasing secretory granules into the lumen of respective organs (postovulatory stalks, ovaries, and oviducts), and eventually ends with the appearance of the homogeneous nutritive fluid. During this process, the height of the epithelial cells gradually and significantly decreased. It remains puzzling what is the mechanism of the transformation of the secretory material from the granular to the fluid form. Future studies are required to answer the questions of whether it is the result of the secretion of enzymes that extracellularly “digest” the released material, or whether secretory granules “dilute” their content due to a liquid transferred from the hemolymph to the lumina of the ovaries and oviducts.

## Conclusions and future perspectives

The results of the study confirm our research hypothesis that in chernetids the secretory phase of the ovarian cycle strongly resembles that described in *Chelifer cancroides*. We clearly showed that chernetids, like *C. cancroides,* are characterized by efficient provisioning the embryos with nutrients produced by three populations of hypertrophic and polyploid epithelial cells, that is the postovulatory stalk cells, the epithelial cell of the ovary and oviduct wall. Our investigations also provide a complete step-by-step description of spectacular alterations that occur in the ovary and oviduct during two consecutive stages of the ovarian cycle, including a new set of data:

i. postovulatory stalks are the most voluminous structures in the ovary and they reach their maximum size when become filled with the nutritive fluid;
ii. secretion of nutrients starts with releasing heterogenous secretory granules, and finishes with diminishing the height of hypertrophic and polyploid cells and transformation of the secretory granules into the homogenous liquid;
iii. polyploid epithelial cells of the ovarian and oviductal wall undergo a massive degeneration after completion of their secretory activity;
iv. epithelium renewal in the ovary and the oviduct occurs due to mitotic activity of the somatic cells that have remained unchanged by hypertrophy and polyploidization.

Although our knowledge of matrotrophy in pseudoscorpions is gradually increasing there are still many gaps and unanswered questions. One of the fundamental questions is how has matrotrophy evolved in this taxon? To address this question, future comparative studies are required to show how the adaptations for matrotrophy look in basal families and the families distantly related to Cheliferidae and Chernetidae.

## Material and methods

49 females of 5 chernetid species (one identified only to genus level) were used in this study, 12 of *Allochernes wideri* (C.L. Koch, 1843), 9 of *Chernes hahnii* (C.L. Koch, 1839), 4 of *Lamprochernes* sp. (Tömösváry, 1883), 16 of *Pselaphochernes lacertosus* (L. Koch, 1873), and 8 of *Pselaphochernes scorpioides* (Hermann, 1804). The specimens of *A. wideri* were collected in spring 2019 in SW Poland, cultured at room temperature and fed with the larvae of a firebrat, *Thermobia domestica* (Packard, 1873) (Insecta: Thysanura: Lepismatidae). The specimens of *C. hahnii, Lamprochernes* sp.*, P. scorpioides* were collected in summer 2020 in Bratislava, Slovakia. The specimens of *P. lacertosus* came from terrarium in Bratislava and were collected in spring 2021.

The vouchers are deposited in the University of Wrocław (Department of Animal Developmental Biology).

### Light and transmission electron microscopy

Whole mount observations of the reproductive system were conducted using an Olympus SZ61 microscope equipped with SC30 camera and Stream Start 1.6.1 software. For histological and ultrastructural observations, the ovaries were dissected and fixed in 2.5% glutaraldehyde in 0.1-M phosphate buffer (pH 7.4) for at least 24 h or longer periods (usually for a few days) at 4 °C. For more details see^27,32^.

### Histochemical analyses

#### Detection of microfilaments, DNA, and plasma membranes

For detection of microfilaments, DNA and plasma membranes the ovaries were fixed in 4% formaldehyde in phosphate-buffered saline (PBS) for 1 h and rinsed in PBS. For detection of microfilaments, the material was stained with 2 mg/ml Alexa Fluor 488 Phalloidin (Invitrogen, A12379). For DNA detection, the material was stained with DAPI (4ʹ,6diamidino-2phenylindole dihydrochloride). For detection of plasma membranes, the ovaries were stained with Wheat Germ Agglutinin Texas Red­X Conjugate (Invitrogen, W21405). For more details see^27^.

#### Detection of endoplasmic reticulum

For detection of endoplasmic reticulum, ovaries were dissected in phosphate ­buffered saline (PBS) and stained with ER-Tracker™ Red (BODIPY™ TR Glibenclamide) (Invitrogen E34250) according to the manufacturer's protocol. After staining, ovaries were rinsed in PBS and whole-mounted onto microscope slides and examined with an Olympus FluoView FV1000 confocal microscope (RRID:SCR_016840).

Images were analysed using GIMP (GNU Image Manipulation Program, RRID:SCR_003182). Figures were prepared in Inkscape (RRID:SCR_014479).

## Data Availability

Data are available on request from the authors.

## References

[1] Ostrovsky AN (2016). Matrotrophy and placentation in invertebrates: A new paradigm. Biol. Rev..

[2] Blackburn DG (1992). Convergent evolution of viviparity, matrotrophy, and specializations for fetal nutrition in reptiles and other vertebrates. Integr. Comp. Biol..

[3] Blackburn DG (2015). Evolution of vertebrate viviparity and specializations for fetal nutrition: A quantitative and qualitative analysis. J. Morphol..

[4] Riesch R, Plath M, Schlupp I, Marsh-Matthews E (2010). Matrotrophy in the cave molly: An unexpected provisioning strategy in an extreme environment. Evol. Ecol..

[5] Ostrovsky AN (2013). From incipient to substantial: Evolution of placentotrophy in a phylum of aquatic colonial invertebrates. Evolution.

[6] Ostrovsky AN, Gordon DP, Lidgard S (2009). Independent evolution of matrotrophy in the major classes of Bryozoa: Transitions among reproductive patterns and their ecological background. Mar. Ecol. Prog. Ser..

[7] Tworzydlo W, Jaglarz MK, Pardyak L, Bilinska B, Bilinski SM (2019). Evolutionary origin and functioning of pregenital abdominal outgrowths in a viviparous insect, *Arixenia esau*. Sci. Rep..

[8] Bilinski SM, Tworzydlo W (2019). Morphogenesis of serial abdominal outgrowths during development of the viviparous dermapteran, *Arixenia esau* (Insecta, Dermaptera). Arthropod Struct. Dev..

[9] Bilinski SM, Sekula M, Tworzydlo W (2020). Morphogenesis of the ovarian follicular epithelium during initial stages of embryogenesis of the viviparous earwig, *Hemimerus talpoides*. J. Morphol..

[10] Akimov IA, Yastrebtsov V (1990). Embryonic development of the mite *Spinturnix vespertiliones* (Parasitiformes: Spinturnicidae). Acarologia.

[11] Yastrebtsov A (1992). Embryonic development of gamasid mites (Parasitiformes: Gamasida). Int. J. Acarol..

[12] Alexeeva N, Tamberg Y (2022). Early lecithotrophic stages of *Nymphon grossipes*, and the role of larval appendages and glands in different larval types of pycnogonids. J. Morphol..

[13] Benavides LR, Cosgrove JG, Harvey MS, Giribet G (2019). Phylogenomic interrogation resolves the backbone of the Pseudoscorpiones tree of life. Mol. Phylogenet. Evol..

[14] Howard RJ, Puttick MN, Edgecombe GD, Lozano-Fernandez J (2020). Arachnid monophyly: Morphological, palaeontological and molecular support for a single terrestrialization within Chelicerata. Arthropod Struct. Dev..

[15] Ontano AZ (2021). Taxonomic sampling and rare genomic changes overcome long-branch attraction in the phylogenetic placement of Pseudoscorpions. Mol. Biol. Evol..

[16] Weygoldt P (1969). The Biology of Pseudoscorpions. Harvard Books in Biology, no. 6.

[17] Anderson, D. T. Chelicerates. In *Embryology and Phylogeny in Annelids and Arthropods* 365–451 (Elsevier, 1973). 10.1016/B978-0-08-017069-5.50014-7.

[18] Makioka T (1968). Morphological and histochemical studies on embryos and ovaries during the embryo-breeding of the pseudoscorpion, *Garypus japonicus*. Sci. Rep. Tokyo Kyoiku Daigaku Sect. B.

[19] Makioka T (1976). Alternative occurrence of two ovarian functions in the adult pseudoscorpion, *Garypus japonicus* Beier. Acta Arachnol..

[20] Makioka T (1979). Structures of the adult ovaries in different functional phases of the pseudoscorpion, *Garypus japonicus* Beier. Acta Arachnol..

[21] Makioka T (1977). The mode of breeding for the embryos and larvae and the breeding stages in the pseudoscorpion, *Garypus japonicus* Beier. Acta Arachnol..

[22] Jędrzejowska I, Maltz TK, Szymusiak K (2019). Formation of the diverticular lumen that enables oocyte fertilization in katoikogenic scorpions, *Heterometrus spinifer* and *Opistophthalmus boehmi* (Scorpionidae). J. Morphol..

[23] Polis, G. A. & Sissom, W. D. Life history. In *The Biology of Scorpions* 161–223 (Stanford Univ Press, 1990).

[24] Chamberlin, J. C. *The arachnid order Chelonethida*. *Stanford university publications. University series. Biological sciences* vol. VII (Stanford University, Calif., Stanford University Press, 1931).

[25] Sareen ML (1965). Histochemical studies on the female germ cells of the pseudoscorpion, *Diplotemnus insolitus* Chamberlin (Chelonetida, Atemnidae). Res. Bull. Panjab Univ..

[26] Dunlop JA (2019). Miniaturisation in chelicerata. Arthropod Struct. Dev..

[27] Jędrzejowska I, Christophoryová J, Garbiec A (2021). Small body size of pseudoscorpions and a distinct architecture of the ovary: A step to miniaturization?. J. Anat..

[28] Makioka, T. Ovarian structure and oogenesis in chelicerates and other arthropods. In *Proceedings of Arthropodan Embryological Society of Japan* 1–10 (1988).

[29] Jędrzejowska, I. Morphology of ovaries and oogenesis in chelicerates. In *Evo-Devo: Non-model Species in Cell and Developmental Biology. Results and Problems in Cell Differentiation* (eds. Tworzydlo, W. & Bilinski, S. M.) Vol. 68, 477–494 (Springer, 2019). 10.1007/978-3-030-23459-1_19 31598868

[30] Badian Z, Ogorzałek A (1982). Fine structure of ovary in *Chelifer cancroides* (Linnaeus, 1761) (Arachnida, Pseudoscorpionidea). Zool. Pol..

[31] Jędrzejowska I, Mazurkiewicz-Kania M, Garbiec A, Kubrakiewicz J (2013). Differentiation and function of the ovarian somatic cells in the pseudoscorpion, *Chelifer cancroides* (Linnaeus, 1761) (Chelicerata: Arachnida: Pseudoscorpionida). Arthropod. Struct. Dev..

[32] Jędrzejowska I, Garbiec A (2020). Adaptations for matrotrophy in the female reproductive system in the pseudoscorpion *Chelifer cancroides* (Chelicerata: Pseudoscorpiones, Cheliferidae). J. Morphol..

[33] Alberti G, Palacios-Vargas JG (2015). Fine structure of the ovary of *Schizomus palaciosi* (Arachnida: Schizomida). Soil Organ..

[34] Alberti G, Gegner A, Witaliński W (1999). Fine structure of the genital system in the females of *Pergamasus* mites (Acari: Gamasida: Pergamasidae). J. Morphol..

[35] Evans GO (1992). Principles of Acarology.

[36] Saito KC (2005). Morphological, histological, and ultrastructural studies of the ovary of the cattle-tick *Boophilus microplus* (Canestrini, 1887) (Acari: Ixodidae). Vet. Parasitol..

[37] Shatrov AB (2002). Oogenesis in ovipositing females of the microtrombidiid mite *Platytrombidium fasciatum* (C.L. Koch) (Acariformes: Microtrombidiidae). Invertebr. Reprod. Dev..

[38] Munson JP (1898). The ovarian egg of *Limulus*. A contribution to the problem of the centrosome and yolk-nucleus. J. Morphol..

[39] Matthiesen FA (1970). Reproductive system and embryos of Brazilian scorpions. An. Acad. Bras. Ciênc..

[40] Francke, O. F. Scorpiones. In *Synopsis and Classification of Living Organisms* (ed. Parker, S. P.) 73–75 (McGraw-Hill, 1982).

[41] Hjelle, J. T. Anatomy and morphology. In *The Biology of Scorpions* (ed. Polis, G. A.) 9–63 (Stanford Univ. Press, 1990).

[42] Klann, A. E. Histology and ultrastructure of solifuges: Comparative studies of organ systems of solifuges (Arachnida, Solifugae) with special focus on functional analyses and phylogenetic interpretations. *Erlangung des akademischen Grades doctor rerum naturalium (Dr. rer. nat.) an der Mathematisch-Naturwissenschaftlichen Fakultät* (2009).

[43] Weygoldt P, Weisemann A, Weisemann K (1972). Morphologisch-histologische Untersuchungen an den Geschlechtsorganen der Amblypygi unter besonderer Berücksichtigung von *Tarantula marginemaculata* C. L. Koch (Arachnida). Zeitschrift für Morphologie der Tiere.

[44] Foelix R (1996). Biology of Spiders.

[45] Weygoldt P (1964). Vergleichend-embryologische Untersuchungen an Pseudoskorpionen (Chelonethi). Z. Morphol. Okol. Tiere.

[46] Moosbrugger M, Schwaha T, Walzl MG, Obst M, Ostrovsky AN (2012). The placental analogue and the pattern of sexual reproduction in the cheilostome bryozoan *Bicellariella ciliata* (Gymnolaemata). Front. Zool..

[47] Lessey, B. A. & Young, S. L. Structure, function, and evaluation of the female reproductive tract. In *Yen and Jaffe’s Reproductive Endocrinology* 206–247.e13 (Elsevier, 2019) 10.1016/B978-0-323-47912-7.00009-3.

[48] Farkaš R (2015). Apocrine secretion: New insights into an old phenomenon. Biochim. Biophys. Acta Gen. Subj..

[49] Farkaš R (2014). Apocrine secretion in *Drosophila* salivary glands: Subcellular origin, dynamics, and identification of secretory proteins. PLoS ONE.

